# Role of Cyclin-Dependent Kinase 1 in Translational Regulation in the M-Phase

**DOI:** 10.3390/cells9071568

**Published:** 2020-06-27

**Authors:** Jaroslav Kalous, Denisa Jansová, Andrej Šušor

**Affiliations:** Institute of Animal Physiology and Genetics, Academy of Sciences of the Czech Republic, Rumburska 89, 27721 Libechov, Czech Republic; Jansova@iapg.cas.cz (D.J.); susor@iapg.cas.cz (A.Š.)

**Keywords:** CDK1, 4E-BP1, mTOR, mRNA, translation, M-phase

## Abstract

Cyclin dependent kinase 1 (CDK1) has been primarily identified as a key cell cycle regulator in both mitosis and meiosis. Recently, an extramitotic function of CDK1 emerged when evidence was found that CDK1 is involved in many cellular events that are essential for cell proliferation and survival. In this review we summarize the involvement of CDK1 in the initiation and elongation steps of protein synthesis in the cell. During its activation, CDK1 influences the initiation of protein synthesis, promotes the activity of specific translational initiation factors and affects the functioning of a subset of elongation factors. Our review provides insights into gene expression regulation during the transcriptionally silent M-phase and describes quantitative and qualitative translational changes based on the extramitotic role of the cell cycle master regulator CDK1 to optimize temporal synthesis of proteins to sustain the division-related processes: mitosis and cytokinesis.

## 1. Introduction

### 1.1. Cyclin Dependent Kinase 1 (CDK1) Is a Subunit of the M Phase-Promoting Factor (MPF)

**Figure 1:**
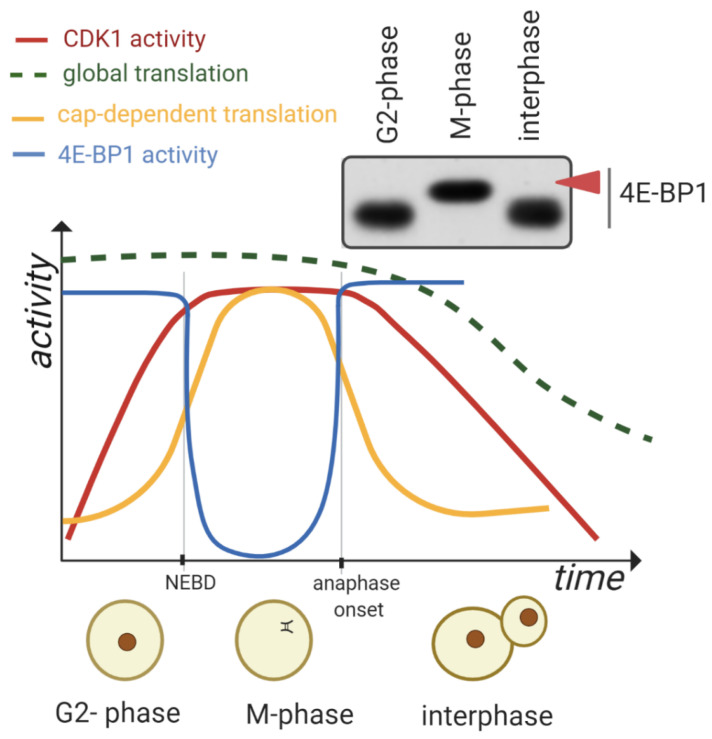
Dynamics of cyclin dependent kinase 1 (CDK1) activity, global translation and inactivation of the translational repressor 4E-BP1 with consequent stimulation of cap-dependent translation during the M-phase. At the beginning of the M-phase, the intensity of global translation is at high levels and CDK1 activity sharply increases accompanied by nuclear envelope breakdown (NEBD). CDK1 activity peaks during assembly of the spindle and 4E-BP1 becomes exclusively hyperphosphorylated (red arrowhead) which results in its inactivation as a translational repressor. At the exit of the M-phase, CDK1 activity drops alongside the increased activity of the cap-dependent translational repressor 4E-BP1. The intensity of global translation decreases during the time course of the M-phase. Immunoblot image using pan 4E-BP1 antibody shows an exclusive phosphorylation shift in the M-phase of the mouse oocyte (red arrowhead) [7].

CDK1, a serine/threonine kinase, is a catalytic subunit of the M phase-promoting factor (MPF) complex which is essential for cell cycle control during the G1-S and G2-M phase transitions of eukaryotic cells. CDK1 is a key player in driving the M-phase in both meiosis and mitosis [1,2]. CDK1 activity sharply increases at the beginning of the M-phase and is inactivated at the end [3,4,5] (Figure 1).

**Figure 2:**
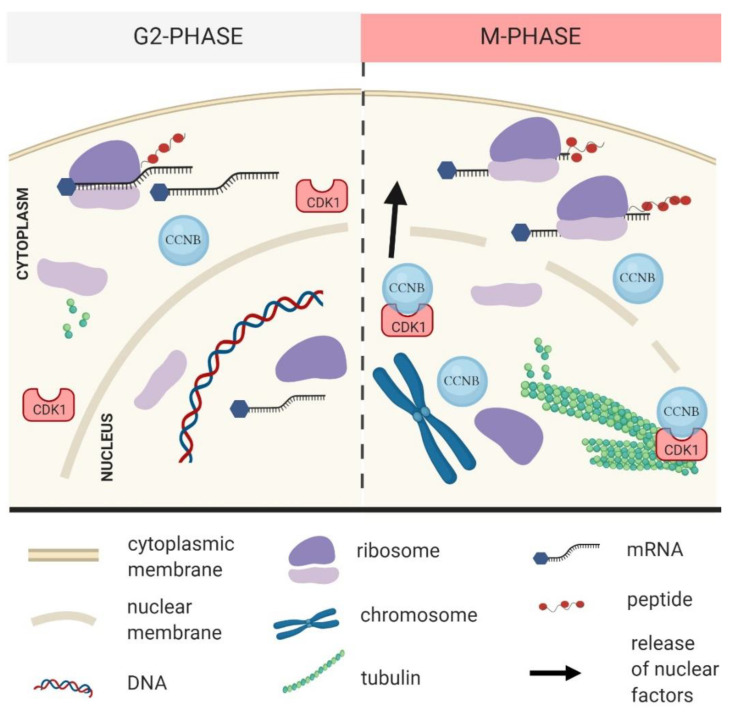
Activated cyclin dependent kinase 1 (CDK1) supports mRNA translation during the M-phase. During the G2-phase, inactive CDK1 with minimal amount of regulatory cyclin B protein (CCNB) is present. Prior to nuclear envelope breakdown (NEBD) CCNB accumulates in the nucleus and binds to CDK1 resulting in an increase of CDK1 activity which leads to NEBD and localization of the complex at the newly forming spindle area. Post NEBD, active CDK1 influences translational reprograming which contributes to successful M-phase progression.

CDK1 is involved in the control of events such as DNA replication and segregation, mRNA transcription, DNA repair and cell morphogenesis (reviewed in [6]). Previous studies identified several translation-associated factors as direct substrates of CDK1 in mitosis and meiosis [7,8]. The association of CDK1 with one of several cyclins is a prerequisite of CDK1 activity (Figure 1 and Figure 2).

In addition to cyclin binding, CDK1 activation requires dephosphorylation on Thr14 and Tyr15 residues and phosphorylation at Thr161 [9,10]. At the onset of the M-phase, cyclin B1 becomes translated and plays a fundamental role in cells entering the M-phase [11]. In oocytes, cyclin B1 and cyclin B2 are involved in the control of the transition from the first to the second meiotic divisions and cyclin B2 compensates for cyclin B1 in CDK1 activation during the M-phase transition in oocyte meiosis [12]. A similar compensatory ability for cyclin B2/CDK1 to interact with separase has been revealed, suggesting that cyclin B2/CDK1 and cyclin B1/CDK1 complexes likely function together in oocytes [13]. The cyclin B3/CDK1 complex is required for the M-phase to anaphase transition in oocytes [14,15].

### 1.2. Global mRNA Translation during the M-Phase

The dynamic control of mRNA translation has a significant impact on many intracellular processes. Translation of mRNA is regulated mostly during the initiation phase by initiation factors interacting with a specific structure bound to the 5′UTR of an mRNA molecule, the 5′ cap (m7GppN) [16,17,18,19]. The cap structure is specifically recognized by the eukaryotic initiation factor complex (eIF4F) comprised of the cap-binding eukaryotic translation initiation factor 4E (eIF4E), the RNA helicase eIF4A and the scaffold protein eIF4G [20,21]. EIF4G also binds the poly(A)-binding protein (PABP) [22], thereby enabling the circularization of the mRNA [23].

**Figure 3:**
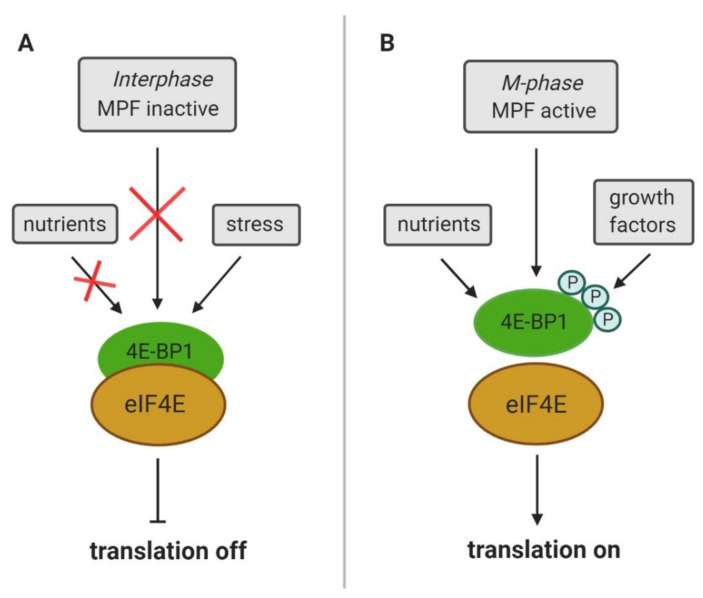
Positive and negative factors affecting eukaryotic translation initiation. (**A**) Inactivity of M phase-promoting factor (MPF) during interphase; lack of nutrients or stress lead to dephosphorylation of eukaryotic translation initiation factor 4E (eIF4E)-binding protein 1 (4E-BP1) which results in formation of translational repressory complex on mRNA. (**B**) Active MPF, availability of nutrients or growth factors lead to hyperphosphorylation of 4E-BP1 resulting in dissociation from eIF4E and promoting formation of 4F complex and translation of specific mRNAs (e.g., mRNAs containing 5′UTR TOP motive).

Suppression of cap-dependent translation coincides with eIF4E dephosphorylation (reviewed in [1]) and occurs together with an increased level of hypophosphorylated 4E-BP1 [24]. The phosphorylation status of 4E-BP1 depends on nutrients, extracellular signals and stress factors (reviewed in [25]) (Figure 3). 

According to the published data, it is considered that global translation is substantially decreased during the M-phase (Figure 1) [26,27,28,29]. Five decades ago a 50–70% reduction of global protein synthesis in synchronized mammalian cells undergoing mitosis was described [30,31] and more recent data presented a 35% decrease in the translation rate during mitosis [29].

It has been documented that the intensity of protein synthesis suppression in cells undergoing mitosis is related to the method of cell synchronization [32,33]. An objection has been raised that the data of downregulation of protein translation in mitotic cells were built on the effects of cellular stress associated with cell cycle synchronization protocol [34]. The reduction of global translation in cells undergoing cell division is believed to result from phosphorylation changes in translation initiation factors. The increased phosphorylation of an α subunit of eukaryotic initiation factor 2α (eIF2α) induced in synchronized cells by cell cycle progression through the G2/M phase was determined as the cause of the downregulation of mRNA translation [35,36]. An intense and long-term eIF2α phosphorylation on Ser51 can suppress global translation and induce cell death [37,38]. When the possible effect of synchronization on translation rates was eliminated, flow cytometry data revealed that there were no significant variations in global translation rates throughout the cell cycle [36].

In contrast to populations of nonsynchronized cells, the oocytes isolated from mammalian ovaries are arrested in the prophase of the first meiotic division and synchronously resume meiosis without chemical induction [39,40]. In oocytes, global translation decreases during meiotic progression and postfertilization [41,42]. Despite CDK1 activity in the M-phase leading to a significant reduction of global translation in mitosis [29] and meiosis [41,42], a subset of mRNAs become translated during the M-phase progression which might be the result of CDK1 influencing the upregulation of the mammalian target of rapamycin (mTOR)/4F axis [7,26,27,43,44]. The above-mentioned data indicate that a decrease in global translation is conserved between mitosis and meiosis. In oocytes from various mammalian species, cap-dependent translation is upregulated after nuclear envelope breakdown (NEBD, Figure 1) and decreased postfertilization [26,27,42,43].

## 2. CDK1 Contributes to the Reprograming of Translation during M-Phase

Ribosomes are considered as executors of the translational program through their role in mRNA decoding and protein synthesis. In eukaryotes, ribosome assembly is a complex process involving more than 200 assembly factors and takes place in the cytoplasm. The activity of CDK1 is coupled with NEBD (Figure 1) leading to the release of a number of nuclear factors which have either a positive or a negative role in the fate of the mRNA e.g., RNA binding proteins, rRNA, ncRNAs and mRNA. Thus CDK1, via nuclear factor release, has direct and indirect influences on translational reprograming as the cell enters M-phase (reviewed in [45]). 

CDK1 regulates ribosome assembly by targeting specific ribosomal proteins. During the G2/M phase, CDK1 phosphorylates ribosomal protein S3 (RPS3), which is a multifunctional protein involved in translation, DNA repair and apoptosis. Phosphorylation of RPS3 by CDK1 is important for the nuclear accumulation of RPS3 [46]. RPS3 is localized evenly in the cytoplasm of germinal vesicle (GV) oocytes and with a higher concentration at the newly forming spindle in NEBD oocytes and mitotic cells [47,48]. 

It was documented that CDK1 cosediments in the polysome fraction and mass spectrometry has revealed that CDK1 is associated with ribosomes [49]. This finding is in line with the notion that ribosomal protein L12 (RPL12) is a known substrate of CDK1 (Table 1) and RPL12 phosphorylation has been shown to enhance the mitotic translation program [50].

RPL12 has been reported to be phosphorylated on Ser38 in different species [57]. Phosphorylation of RPL12 regulates the translation of specific subsets of RNA during mitosis [50]. In HeLa cells, phosphorylation of RPL12 at Ser38 peaks in mitosis and reaches the lowest level during the S-phase [50,58]. In eukaryotes, the sequence surrounding Ser38 is highly conserved and matches a consensus motif for CDK1 substrates [50]. Inhibition of CDK1 induced a progressive decrease in the percentage of heavy polysomes and a decline in polypeptide synthesis [13]. 

The nucleolus is a large nuclear organelle assembled around ribosomal genes (rDNAs) and is a site of ribosomal assembly. The assembly and disassembly of the nucleolus depends on the equilibrium between the phosphorylation/dephosphorylation of the transcription machinery and on the preribosomal ribonucleoprotein (RNP) complexes processing under the control of CDK1 and PP1 phosphatases [59]. CDK1 regulates the activity of the nucleolar phosphoprotein nucleophosmin/B23 involved in the regulation of rRNA transcription through histone chaperone activity [60]. B23/nucleophosmin associates with rRNA chromatin to stimulate rRNA transcription [61]. It has been shown that B23/nucleophosmin interacts with maturing ribosomal subunits and is involved in their nuclear export [62]. During mitosis, phosphorylation of B23/nucleophosmin by CDK1 at specific sites induces a release of B23/nucleophosmin from chromatin and inactivates its RNA binding activity [61,63].

## 3. CDK1 Activity and Localization in the Cell

One insight into how a single kinase can coordinate so many different events is that cyclin B1-CDK1 is targeted to different structures as the cell enters meiosis/mitosis. The key activator of CDK1, cyclin B [64,65], is activated on centrosomes [66] and a large fraction immediately moves into the nucleus preceding the breakdown of the nuclear envelope [67,68]. Subsequently, Cyclin B1-CDK1 binds to the microtubules, chromosomes and to unattached kinetochores in the prometaphase [7,69]. These observations indicate that the localization of Cyclin B1-CDK1 may be an important determinant of how specific substrates are recognized at specific times. In connection, we previously detected by in situ translation that distinct translational areas are present on the newly forming spindle and chromosomes in the post NEBD oocyte which are influenced by the mTOR/4F axis [8,17]. Concomitant CDK1 activation and NEBD leads to the release of nuclear factors which might be partly retained in the spindle area by a semipermeable membrane containing lamins, endoplasmic reticulum and mitochondria which may function in oocyte spindle assembly as an organelle-exclusion spindle envelope [27,70,71]. Localization of CDK1 at the spindle area leads to the inactivation of the translational repressor 4E-BP1 [7,8,33]. Thus, localization of CDK1 and its direct negative influence on a crucial translational repressor stimulate localized translation in the newly forming spindle to promote spindle architecture and faithful chromosome segregation.

## 4. CDK1 Substitutes for mTOR Control of Cap-Dependent Translation

**Figure 4:**
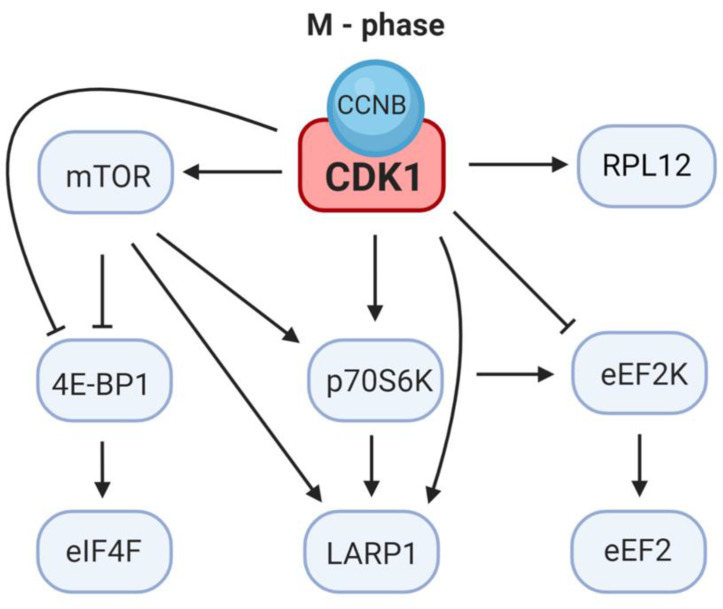
Schematic representation of direct extramitotic effect of cyclin dependent kinase 1 (CDK1) on molecular axis influencing mRNA translation during M-phase. CDK1 activates the mammalian target of rapamycin (mTOR) and subsequently mTOR phosphorylates and activates its substrates ribosomal protein S6 kinase B1 (p70S6K) and LA-related protein 1 (LARP1). Phosphorylation of eukaryotic translation initiation factor 4E (eIF4E)-binding protein 1 (4E-BP1) by mTOR leads to the dissociation of 4E-BP1 from eIF4E and to the activation of the eIF4F complex. CDK1 directly phosphorylates the mTOR substrates 4E-BP1, p70S6K and LARP1. CDK1 exerts inhibitory phosphorylation of the eukaryotic elongation factor 2 kinase (eEF2K) and, as a consequence, the activity of elongation factor 2 (eEF2) is stimulated. The activity of ribosomal protein L12 (RPL12) is also CDK1-dependent.

mTOR, a serine/threonine protein kinase, regulates diverse cellular functions (reviewed in [72]) and mTOR clearly contributes to the translation of both TOP and nonTOP mRNAs (reviewed in [73]). Besides CDK1, mTOR is the main regulator of 4E-BP1 activity (Figure 3, Figure 4) [73], thereby releasing eIF4E and activating translation [74]. Upon inhibition of mTOR, 4E-BP1 is dephosphorylated and the affinity of 4E-BP1 for eIF4E increases [75]. mTOR enhances the translation of mRNAs containing the 5′ terminal oligopyrimidine (5′ TOP) motif [76,77]. It has been documented that overexpression of a rapamycin-resistant mTOR mutant restored the rapamycin-inhibited activity of mTOR effectors ribosomal protein S6 kinase B1 (p70S6K) and 4E-BP1 and removed rapamycin-induced inhibition of cell cycle progression [78]. Although mTOR is considered to be the main kinase phosphorylating 4E-BP1, several other kinases are involved in the phosphorylation of various 4E-BP1 residues (reviewed in [74]). 

Phosphorylation of the main mTOR substrates, p70S6K and 4E-BP1, is also dependent on CDK1 during mitosis [8,13,33] (Table 1) and CDK1 can substitute for mTOR kinase in the activation of cap-dependent translation in mitotic cells, suggesting that an alternate pathway for the regulation of cap-dependent translation exists [29,33]. It has been observed in mouse prophase lymphoblasts that CDK1 promotes mitotic growth through the increased phosphorylation of 4E-BP1 and cap-dependent protein synthesis [79].

When 4E-BP1 is hyperphosphorylated at Thr37/Thr46, Thr70 and Ser65, the eIF4G:eIF4E interaction is enabled and the initiation of cap-dependent translation begins (reviewed in [24]). It has been proposed that phosphorylation of 4E-BP1 at Thr37/Thr46 is rather constitutive, and phosphorylation at Ser65 is more related to its effect on cap-dependent translation [59,80]. Although the priming phosphorylation sites Thr-37/Thr-46 of 4E-BP1 are targeted by mTOR [81], the Thr37/Thr46 residues can also be phosphorylated by CDK1 [33]. CDK1 also phosphorylates 4E-BP1 at the Thr70 residue, which is one of several phosphorylation sites required for the inactivation of 4E-BP1 [7,8]. It has been suggested that mTOR may act in concert with CDK1 to generate fully inactivated/phosphorylated 4E-BP1 isoforms during mitosis [24]. 

Increased phosphorylation of 4E-BP1 occurs during the meiotic M-phase in porcine, bovine and mouse oocytes [26,27,42,82]. Although it is also diffusely located in the oocyte cytoplasm, the presence of phosphorylated 4E-BP1 on the meiotic spindle poles, kinetochores and along the polar microtubules suggests that it represents a possible means of supporting spatially localized protein production [82]. CDK1 and mTOR are the main positive regulators of 4E-BP1 phosphorylation during meiosis in mouse oocytes and CDK1 affects the activity of mTOR localized in the vicinity of the chromosomes and on the MII spindle, suggesting that CDK1 acts indirectly on 4E-BP1 phosphorylation via mTOR activation [7]. 

It can be concluded that the phosphorylation of 4E-BP1 promotes translation during the M-phase to support spindle assembly, highlighting the importance of the role of CDK1 and mTOR kinase in this process. 

During the M-phase CDK1 also phosphorylates and inactivates p70S6K, the other mTOR substrate [83,84]. It has been proposed that p70S6K is involved in the regulation of TOP mRNA translation because the substrates of p70S6K include the eukaryotic translation elongation factor 1A (eEF1A), eukaryotic elongation factor 2 (eEF2) and several ribosomal proteins [76]. Therefore, CDK1 activity could be involved in the regulation of translation during the M-phase by decreasing the amount of translation factors available. Although p70S6K is phosphorylated by CDK1 at several sites, CDK1 does not phosphorylate p70S6K at Thr389, the mTOR phosphorylation site [84]. In HeLa cells the inhibition of CDK1 resulted in reduced phosphorylation of the ribosomal protein S6 (RPS6), a p70S6K substrate, indicating that CDK1 supports the initiation of translation through the p70S6K signaling pathway. However, CDK1 likely does not act via mTOR, as CDK1 inhibition did not alter the integrity of the cap binding complex [13]. 

## 5. CDK1 Regulates the Elongation Step of Translation

In eukaryotes, the culmination of translation initiation coincides with the formation of an 80S initiation complex in which initiator methionine transfer RNA (Met-tRNAi^Met^) is bound to the P (peptidyl) site of the ribosome. The anticodon of the Met-tRNAi^Met^ is base-paired with the start codon of the mRNA, and the second codon of the open reading frame (ORF) is localized on the A (aminoacyl) site of the ribosome. Elongation is initiated when the cognate elongating aminoacyl-tRNA is delivered to the A site of the ribosome. The eukaryotic translation elongation factor eEF1A is activated upon binding to GTP and creates a ternary complex upon binding to aminoacyl-tRNA [54,85].

CDK1 modulates the activity of elongation factors (Table 1). Translation elongation in eukaryotes is mediated by the determined actions of elongation factor 1A (eEF1A); elongation factor 1B (eEF1B) complex and elongation factor 2 (eEF2). In HeLa cells, and in sea urchin embryos, the translation elongation rate declines in synchrony with an increase of CDK1 activity [86,87]. The eEF2 kinase (eEF2K), which inactivates eEF2, is inhibited by phosphorylation at Ser359 and CDK1 has been identified as the kinase phosphorylating eEF2K at Ser359, indicating that CDK1 stimulates the activity of eEF2 during mitosis [56].

In human cells, a conserved consensus phosphorylation site for mitotic CDK1 is present on the catalytic δ subunit of eEF1B (termed “eEF1D”). The eEF1D and eEF1Bγ subunits are physiological substrates for CDK1 during the resumption of meiosis in *Xenopus* oocytes [55,88].

In monkey kidney epithelial cells, two eEF1D consensus CDK1 target sites were identified, Ser-133 and Thr-147, whilst eEF1D has been reported to be phosphorylated by CDK1 on Ser-133 in vitro [89]. Phosphorylation of Ser-133 during mitosis in HeLa cells is necessary for the reduced interaction of eEF1D with its substrate eEF1A and leads to a slowdown of translation elongation [54]. It has been proposed that phosphorylation of eEF1D by CDK1 leads to reduced interaction of the catalytic subunit eEF1D with its substrate eEF1A·GDP causing a decrease in the guanine nucleotide exchange rate by the eEF1B complex followed by a lower level of active eEF1A·GTP during mitosis. Subsequently, less eEF1A·GTP is available for binding and delivering aa-tRNA to ribosomes, inducing a translational slowdown [54]. The above-mentioned data provide clear evidence of the importance of CDK1 in the regulation of the elongation step of translation in eukaryotes.

## 6. CDK1 Modulates LARP1 Activity

CDK1 has been identified to phosphorylate the evolutionarily conserved RNA binding protein, LA-related protein 1 (LARP1) [13]. Recent studies revealed that LARP1 plays a key role in protein synthesis, as it was shown to regulate both the stability and translation of mRNAs characterized by a 5′TOP motif [90,91]. LARP1 was identified as a potential mTOR complex 1 (mTORC1) substrate [92,93] and LARP1 was shown to associate with the 3′ end of TOP mRNAs via binding with poly(A) binding protein (PABP) [91,94]. LARP1-bound mRNAs reveal that nonphosphorylated LARP1 interacts with both 5′ and 3′UTRs of RP mRNAs and inhibits their translation [91]. Phosphorylation of LARP1 dissociates it from 5′UTRs and reduces its inhibitory activity to facilitate synthesis of ribosomal proteins, a subclass of TOP mRNAs. Thus, in response to CDK1 and mTOR activity, LARP1 serves as a phosphorylation-sensitive molecular switch for turning on TOP mRNA translation and subsequent ribosome biogenesis. Concomitantly, phosphorylated LARP1 scaffolds mTORC1 on the 3′UTRs of translationally-competent TOP mRNAs [91]. Depletion of LARP1 leads to mitotic arrest with a detectable increase in the level of cyclin B1 [95]. The effect of LARP1 depletion appears to be reduced cell growth and proliferation [90,94], which is consistent with the activity of CDK1. In connection with this, LARP1 was found to be significantly upregulated in several malignancies [95,96,97,98]. These findings suggest that elevated LARP1 expression might be linked to cellular proliferation.

## 7. Perspectives

Here we summarize findings that point to an extramitotic role of CDK1 in the cell to couple M-phase progression with protein expression. The challenge here is to experimentally uncouple the timing of M-phase progression from the extramitotic function of CDK1. Novel methods such as e.g., imaging and next generation sequencing (NGS) should be applied to the naturally cycling cells to further examine these tightly coupled cellular processes. An interesting feature of CDK1-cyclin B is its localization in the cell, which might be involved in the regulation of various substrates and the spatiotemporal coupling of two conserved molecular modules, CDK1-cyclin B for the cell cycle and translational regulation for gene expression. This might shed light on the physiological role of localized translation in the newly forming spindle. Future research is needed to elucidate more precisely the role of CDK1 in the regulation of mRNA translation. Next generation sequencing of the polysomal fractions and cutting-edge proteomics should be used to identify the positive and negative translational changes. Additionally, detailed genome-wide analyses might reveal a subclass of transcriptome, or their regulatory motives, which are specifically influenced by CDK1 activity.

The direct effect of CDK1 activity on translational regulation is difficult to pinpoint because experimental manipulation of CDK1 activity might influence the timing of the cell cycle progression and, moreover, a number of CDK1 substrates might play an intermediate role in this process. We expect that more and more substrates with direct/indirect roles in protein synthesis will emerge in the coming years.

Further research should be oriented to the extramitotic functioning of CDK1 in the regulation of translation. Description of the broader role of CDK1 will provide a new insight into the specialized translation and translational control in human diseases such as cancer. Dysregulation of CDK1, which leads to increased cell proliferation, has been identified in various cancers. In connection with this, LARP1 as a substrate of CDK1 is upregulated in certain cancer types, which leads to an elevated survival rate probably via aberrant translation. Accordingly, the regulation of CDK1 and the usage of CDK-inhibitors have provided encouraging results in the treatment of cancer.

## Figures and Tables

**Table 1:** Known cyclin dependent kinase 1 (CDK1) substrates involved in translation. CDK1 controls the phosphorylation status and activity of proteins involved in the initiation (mammalian target of rapamycin (mTOR), eukaryotic translation initiation factor 4E (eIF4E)-binding protein 1 (4E-BP1), ribosomal protein S6 kinase B1 (p70S6K)) and elongation (eukaryotic elongation factor 2 kinase (eEF2K)) steps of mRNA translation.

	CDK1 Substrate	Ref.	Role of CDK1 Substrate
CDK1 and mTOR	Raptor	[51]	mTOR phosphorylation and activation
	mTOR (Ser2448)	[7]	4E-BP1 and p70S6K phosphorylation
	p70S6K	[8,33]	Regulation of TOP mRNA translation
	LARP1	[13,52]	Regulation of TOP mRNA translation
CDK1 and 4E-BP1	4E-BP1 (Thr37/Thr46)	[33]	Thr37/Thr46 phosphorylation primes 4E-BP1 for Ser65 and Thr70 phosphorylation
	4E-BP1 (Thr70)	[7,8]	Thr70 phosphorylation regulates 4E-BP1
	4E-BP1 (Ser83)	[8]	The role of Ser83 phosphorylation is unknown
CDK1 and ribo-some assembly	eIF2α	[13]	Regulation of eIF2 complex
	eIF4G1	[53]	mRNA recruitment to the ribosome
	RPL12	[50]	Translational program
CDK1 and elongation	eEF1B	[54]	Allows ribosome movement along RNA
	eEF1D	[55]	Delivery of aminoacyl-tRNA to the ribosome
	eEF2K	[56]	Regulation the elongation step of protein synthesis

## References

[1] Adhikari D., Zheng W., Shen Y., Gorre N., Ning Y., Halet G., Kaldis P., Liu K. (2012). Cdk1, but not Cdk2, is the sole Cdk that is essential and sufficient to drive resumption of meiosis in mouse oocytes. Hum. Mol. Genet..

[2] Diril M.K., Ratnacaram C.K., Padmakumar V.C., Du T., Wasser M., Coppola V., Tessarollo L., Kaldis P. (2012). Cyclin-dependent kinase 1 (Cdk1) is essential for cell division and suppression of DNA re-replication but not for liver regeneration. Proc. Natl. Acad. Sci. USA.

[3] Dorée M., Peaucellier G., Picard A. (1983). Activity of the maturation-promoting factor and the extent of protein phosphorylation oscillate simultaneously during meiotic maturation of starfish oocytes. Dev. Biol..

[4] Picard A., Labbe J.C., Doree M. (1988). The cell cycle can occur in starfish oocytes and embryos without the production of transferable MPF (maturation-promoting factor). Dev. Biol..

[5] Wasserman W., Masui Y. (1975). Effects of cycloheximide on a cytoplasmic factor initiating meiotic maturation in Xenopus oocytes. Exp. Cell Res..

[6] Enserink J.M., Kolodner R.D. (2010). An overview of Cdk1-controlled targets and processes. Cell Div..

[7] Jansova D., Koncicka M., Tetkova A., Cerna R., Malik R., del Llano E., Kubelka M., Susor A. (2017). Regulation of 4E-BP1 activity in the mammalian oocyte. Cell Cycle.

[8] Velásquez C., Cheng E., Shuda M., Lee-Oesterreich P.J., Von Strandmann L.P., Gritsenko M.A., Jacobs J.M., Moore P.S., Chang Y. (2016). Mitotic protein kinase CDK1 phosphorylation of mRNA translation regulator 4E-BP1 Ser83 may contribute to cell transformation. Proc. Natl. Acad. Sci. USA.

[9] Krek W., Nigg E.A. (1991). Mutations of p34cdc2 phosphorylation sites induce premature mitotic events in HeLa cells: Evidence for a double block to p34cdc2 kinase activation in vertebrates. EMBO J..

[10] Solomon M.J., Glotzer M., Lee T.H., Philippe M., Kirschner M.W. (1990). Cyclin activation of p34cdc2. Cell.

[11] Tachibana K., Ishiura M., Uchida T., Kishimoto T. (1990). The starfish egg mRNA responsible for meiosis reinitiation encodes cyclin. Dev. Biol..

[12] Li J., Tang J.X., Cheng J.M., Hu B., Wang Y.Q., Aalia B., Li X.Y., Jin C., Wang X.X., Deng S.L. (2018). Cyclin B2 can compensate for Cyclin B1 in oocyte meiosis I. J. Cell Biol..

[13] Haneke K., Schott J., Lindner D., Hollensen A.K., Damgaard C.K., Mongis C., Knop M., Palm W., Ruggieri A., Stoecklin G. (2020). CDK1 couples proliferation with protein synthesis. J. Cell Biol..

[14] Li Y., Wang L., Zhang L., He Z., Feng G., Sun H., Wang J., Li Z., Liu C., Han J. (2019). Cyclin b3 is required for metaphase to anaphase transition in oocyte meiosis I. J. Cell Biol..

[15] Karasu M.E., Bouftas N., Keeney S., Wassmann K. (2019). Cyclin B3 promotes anaphase i onset in oocyte meiosis. J. Cell Biol..

[16] Kozak M. (1978). How do eucaryotic ribosomes select initiation regions in messenger RNA?. Cell.

[17] Merrick W.C., Pavitt G.D. (2018). Protein synthesis initiation in eukaryotic cells. Cold Spring Harb. Perspect. Biol..

[18] Shirokikh N.E., Preiss T. (2018). Translation initiation by cap-dependent ribosome recruitment: Recent insights and open questions. Wiley Interdiscip. Rev. RNA.

[19] Hinnebusch A.G., Ivanov I.P., Sonenberg N. (2016). Translational control by 5′-untranslated regions of eukaryotic mRNAs. Science.

[20] Imataka H., Gradi A., Sonenberg N. (1998). A newly identified N-terminal amino acid sequence of human eIF4G binds poly(A)-binding protein and functions in poly(A)-dependent translation. EMBO J..

[21] Sonenberg N., Morgan M.A., Merrick W.C., Shatkin A.J. (1978). A polypeptide in eukaryotic initiation factors that crosslinks specifically to the 5′-terminal cap in mRNA. Proc. Natl. Acad. Sci. USA.

[22] Sachs A.B., Davis R.W. (1989). The poly(A) binding protein is required for poly(A) shortening and 60S ribosomal subunit-dependent translation initiation. Cell.

[23] Wells S.E., Hillner P.E., Vale R.D., Sachs A.B. (1998). Circularization of mRNA by eukaryotic translation initiation factors. Mol. Cell.

[24] Sun R., Cheng E., Velásquez C., Chang Y., Moore P.S. (2019). Mitosis-related phosphorylation of the eukaryotic translation suppressor 4E-BP1 and its interaction with eukaryotic translation initiation factor 4E (eIF4E). J. Biol. Chem..

[25] Sengupta C., Peterson T.R., Sabatini D.M., Sengupta S. (2010). Regulation of the mTOR Complex 1 pathway by nutrients, growth factors, and stress. Mol. Cell.

[26] Ellederova Z., Kovarova H., Melo-Sterza F., Livingstone M., Tomek W., Kubelka M. (2006). Suppression of translation during in vitro maturation of pig oocytes despite enhanced formation of cap-binding protein complex eIF4F and 4E-BP1 hyperphosphorylation. Mol. Reprod. Dev..

[27] Susor A., Jansova D., Cerna R., Danylevska A., Anger M., Toralova T., Malik R., Supolikova J., Cook M.S., Oh J.S. (2015). Temporal and spatial regulation of translation in the mammalian oocyte via the mTOR-eIF4F pathway. Nat. Commun..

[28] Pyronnet S., Dostie J., Sonenberg N. (2001). Suppression of cap-dependent translation in mitosis. Genes Dev..

[29] Tanenbaum M.E., Stern-Ginossar N., Weissman J.S., Vale R.D. (2015). Regulation of mRNA translation during mitosis. eLife.

[30] Fan H., Penman S. (1970). Regulation of protein synthesis in mammalian cells. II. Inhibition of protein synthesis at the level of initiation during mitosis. J. Mol. Biol..

[31] Tarnowka M.A., Baglioni C. (1979). Regulation of protein synthesis in mitotic HeLa cells. J. Cell. Physiol..

[32] Coldwell M.J., Cowan J.L., Vlasak M., Mead A., Willett M., Perry L.S., Morley S.J. (2013). Phosphorylation of eIF4GII and 4E-BP1 in response to nocodazole treatment: A reappraisal of translation initiation during mitosis. Cell Cycle.

[33] Shuda M., Velásquez C., Cheng E., Cordek D.G., Kwun H.J., Chang Y., Moore P.S. (2015). CDK1 substitutes for mTOR kinase to activate mitotic cap-dependent protein translation. Proc. Natl. Acad. Sci. USA.

[34] Anda S., Grallert B. (2019). Cell-Cycle-Dependent Regulation of Translation: New interpretations of old observations in light of new approaches. BioEssays.

[35] Silva R.C., Dautel M., Di Genova B.M., Amberg D.C., Castilho B.A., Sattlegger E. (2015). The Gcn2 Regulator Yih1 Interacts with the cyclin dependent kinase Cdc28 and promotes cell cycle progression through G2/M in budding yeast. PLoS ONE.

[36] Stonyte V., Boye E., Grallert B. (2018). Regulation of global translation during the cell cycle. J. Cell Sci..

[37] Uppala J.K., Ghosh C., Sathe L., Dey M. (2018). Phosphorylation of translation initiation factor eIF2α at Ser51 depends on site- and context-specific information. FEBS Lett..

[38] Gordiyenko Y., Llácer J.L., Ramakrishnan V. (2019). Structural basis for the inhibition of translation through eIF2α phosphorylation. Nat. Commun..

[39] De La Fuente R., Viveiros M.M., Burns K.H., Adashi E.Y., Matzuk M.M., Eppig J.J. (2004). Major chromatin remodeling in the germinal vesicle (GV) of mammalian oocytes is dispensable for global transcriptional silencing but required for centromeric heterochromatin function. Dev. Biol..

[40] Eppig J.J., Schroeder A.C. (1989). Capacity of Mouse Oocytes from Preantral Follicles to Undergo Embryogenesis and Development to Live Young after Growth, Maturation, and Fertilization in Vitro1. Biol. Reprod..

[41] Šušor A., Jelínková L., Karabínová P., Torner H., Tomek W., Kovářová H., Kubelka M. (2008). Regulation of cap-dependent translation initiation in the early stage porcine parthenotes. Mol. Reprod. Dev..

[42] Ellederová Z., Cais O., Šušor A., Uhlířová K., Kovářová H., Jelínková L., Tomek W., Kubelka M. (2008). ERK1/2 map kinase metabolic pathway is responsible for phosphorylation of translation initiation factor eIF4E during in vitro maturation of pig oocytes. Mol. Reprod. Dev..

[43] Tomek W., Sterza F.A.M., Kubelka M., Wollenhaupt K., Torner H., Anger M., Kanitz W. (2002). Regulation of Translation During In Vitro Maturation of Bovine Oocytes: The Role of MAP Kinase, eIF4E (Cap Binding Protein) Phosphorylation, and eIF4E-BP11. Biol. Reprod..

[44] Ramírez-Valle F., Badura M.L., Braunstein S., Narasimhan M., Schneider R.J. (2010). Mitotic Raptor Promotes mTORC1 Activity, G2/M Cell Cycle Progression, and Internal Ribosome Entry Site-Mediated mRNA Translation. Mol. Cell. Biol..

[45] Fromont-Racine M., Senger B., Saveanu C., Fasiolo F. (2003). Ribosome assembly in eukaryotes. Gene.

[46] Yoon I.S., Chung J.H., Hahm S.H., Park M.J., Lee Y.R., Ko S.I., Kang L.W., Kim T.S., Kim J., Han Y.S. (2011). Ribosomal protein S3 is phosphorylated by Cdk1/cdc2 during G2/M phase. BMB Rep..

[47] Susor A., Kubelka M. (2017). Translational regulation in the mammalian oocyte. Results and Problems in Cell Differentiation.

[48] Jang C.Y., Kim H.D., Zhang X., Chang J.S., Kim J. (2012). Ribosomal protein S3 localizes on the mitotic spindle and functions as a microtubule associated protein in mitosis. Biochem. Biophys. Res. Commun..

[49] Simsek D., Tiu G.C., Flynn R.A., Byeon G.W., Leppek K., Xu A.F., Chang H.Y., Barna M. (2017). The Mammalian Ribo-interactome Reveals Ribosome Functional Diversity and Heterogeneity. Cell.

[50] Imami K., Milek M., Bogdanow B., Yasuda T., Kastelic N., Zauber H., Ishihama Y., Landthaler M., Selbach M. (2018). Phosphorylation of the Ribosomal Protein RPL12/uL11 Affects Translation during Mitosis. Mol. Cell.

[51] Odle R.I., Walker S.A., Oxley D., Kidger A.M., Balmanno K., Gilley R., Okkenhaug H., Florey O., Ktistakis N.T., Cook S.J. (2020). An mTORC1-to-CDK1 Switch Maintains Autophagy Suppression during Mitosis. Mol. Cell.

[52] Berman A.J., Thoreen C.C., Dedeic Z., Chettle J., Roux P.P., Sarah B.P. (2020). Controversies around the function of LARP1. RNA Biol..

[53] Dobrikov M.I., Shveygert M., Brown M.C., Gromeier M. (2014). Mitotic Phosphorylation of Eukaryotic Initiation Factor 4G1 (eIF4G1) at Ser1232 by Cdk1:Cyclin B Inhibits eIF4A Helicase Complex Binding with RNA. Mol. Cell. Biol..

[54] Sivan G., Aviner R., Elroy-Stein O. (2011). Mitotic modulation of translation elongation factor 1 leads to hindered tRNA delivery to ribosomes. J. Biol. Chem..

[55] Mulner-Lorillon O., Minella O., Cormier P., Capony J.P., Cavadore J.C., Morales J., Poulhe R., Bellé R. (1994). Elongation factor EF-1 delta, a new target for maturation-promoting factor in Xenopus oocytes. J. Biol. Chem..

[56] Smith E.M., Proud C.G. (2008). cdc2-cyclin B regulates eEF2 kinase activity in a cell cycle and amino acid-dependent manner. EMBO J..

[57] Gnad F., Gunawardena J., Mann M. (2011). PHOSIDA 2011: The posttranslational modification database. Nucleic Acids Res..

[58] Olsen J.V., Vermeulen M., Santamaria A., Kumar C., Miller M.L., Jensen L.J., Gnad F., Cox J., Jensen T.S., Nigg E.A. (2010). Quantitative phosphoproteomics revealswidespread full phosphorylation site occupancy during mitosis. Sci. Signal..

[59] Hernandez-Verdun D. (2011). Assembly and disassembly of the nucleolus during the cell cycle. Nucleus.

[60] Murano K., Okuwaki M., Hisaoka M., Nagata K. (2008). Transcription regulation of the rRNA gene by a multifunctional nucleolar protein, B23/nucleophosmin, through its histone chaperone activity. Mol. Cell. Biol..

[61] Okuwaki M., Matsumoto K., Tsujimoto M., Nagata K. (2001). Function of nucleophosmin/B23, a nucleolar acidic protein, as a histone chaperone. FEBS Lett..

[62] Yu Y., Maggi L.B., Brady S.N., Apicelli A.J., Dai M.-S., Lu H., Weber J.D. (2006). Nucleophosmin is essential for ribosomal protein L5 nuclear export. Mol. Cell. Biol..

[63] Hisaoka M., Ueshima S., Murano K., Nagata K., Okuwaki M. (2010). Regulation of nucleolar chromatin by B23/nucleophosmin jointly depends upon its RNA binding activity and transcription factor UBF. Mol. Cell. Biol..

[64] Hagting A., Jackman M., Simpson K., Pines J. (1999). Translocation of cyclin B1 to the nucleus at prophase requires a phosphorylation-dependent nuclear import signal. Curr. Biol..

[65] Lindqvist A., van Zon W., Karlsson Rosenthal C., Wolthuis R.M.F. (2007). Cyclin B1–Cdk1 activation continues after centrosome separation to control mitotic progression. PLoS Biol..

[66] Jackman M., Lindon C., Niggt E.A., Pines J. (2003). Active cyclin B1-Cdk1 first appears on centrosomes in prophase. Nat. Cell Biol..

[67] Gavet O., Pines J. (2010). Progressive activation of CyclinB1-Cdk1 coordinates entry to mitosis. Dev. Cell.

[68] Karabinova P., Kubelka M., Susor A. (2011). Proteasomal degradation of ubiquitinated proteins in oocyte meiosis and fertilization in mammals. Cell Tissue Res..

[69] Pines J., Hunter T. (1991). Cyclin-dependent kinases: A new cell cycle motif?. Trends Cell Biol..

[70] Koncicka M., Tetkova A., Jansova D., Del Llano E., Gahurova L., Kracmarova J., Prokesova S., Masek T., Pospisek M., Bruce A.W. (2018). Increased expression of maturation promoting factor components speeds up meiosis in oocytes from aged females. Int. J. Mol. Sci..

[71] Schweizer N., Pawar N., Weiss M., Maiato H. (2015). An organelle-exclusion envelope assists mitosis and underlies distinct molecular crowding in the spindle region. J. Cell Biol..

[72] Wang X., Proud C.G. (2011). mTORC1 Signaling: What We Still Don’t Know. J. Mol. Cell Biol..

[73] Truitt M.L., Ruggero D. (2016). New frontiers in translational control of the cancer genome. Nat. Rev. Cancer.

[74] Qin X., Jiang B., Zhang Y. (2016). 4E-BP1, a multifactor regulated multifunctional protein. Cell Cycle.

[75] Sonenberg N., Hinnebusch A.G. (2009). Regulation of translation initiation in eukaryotes: Mechanisms and biological targets. Cell.

[76] Meyuhas O., Kahan T. (2015). The race to decipher the top secrets of TOP mRNAs. Biochim. Biophys. Acta.

[77] Yamashita R., Suzuki Y., Takeuchi N., Wakaguri H., Ueda T., Sugano S., Nakai K. (2008). Comprehensive detection of human terminal oligo-pyrimidine (TOP) genes and analysis of their characteristics. Nucleic Acids Res..

[78] Fingar D.C., Richardson C.J., Tee A.R., Cheatham L., Tsou C., Blenis J. (2004). mTOR controls cell cycle progression through its cell growth effectors S6K1 and 4E-BP1/Eukaryotic translation initiation factor 4E. Mol. Cell. Biol..

[79] Miettinen T.P., Kang J.H., Yang L.F., Manalis S.R. (2019). Mammalian cell growth dynamics in mitosis. eLife.

[80] Livingstone M., Bidinosti M. (2012). Rapamycin-insensitive mTORC1 activity controls eIF4E:4E-BP1 binding. F1000Research.

[81] Burnett P.E., Barrow R.K., Cohen N.A., Snyder S.H., Sabatini D.M. (1998). RAFT1 phosphorylation of the translational regulators p70 S6 kinase and 4E-BP1. Proc. Natl. Acad. Sci. USA.

[82] Romasko E.J., Amarnath D., Midic U., Latham K.E. (2013). Association of maternal mRNA and phosphorylated EIF4EBP1 variants with the spindle in mouse oocytes: Localized translational control supporting female meiosis in mammals. Genetics.

[83] Papst P.J., Sugiyama H., Nagasawa M., Lucas J.J., Maller J.L., Terada N. (1998). Cdc2-cyclin B phosphorylates p70 S6 kinase on Ser411 at mitosis. J. Biol. Chem..

[84] Shah O.J., Ghosh S., Hunter T. (2003). Mitotic regulation of ribosomal S6 kinase 1 involves Ser/Thr, Pro phosphorylation of consensus and non-consensus sites by Cdc2. J. Biol. Chem..

[85] Jakobsson M.E., Małecki J., Falnes P. (2018). Regulation of eukaryotic elongation factor 1 alpha (eEF1A) by dynamic lysine methylation. RNA Biol..

[86] Sivan G., Elroy-Stein O. (2008). Regulation of mRNA Translation during cellular division. Cell Cycle.

[87] Monnier A., Bellé R., Morales J., Cormier P., Boulben S., Mulner-Lorillon O. (2001). Evidence for regulation of protein synthesis at the elongation step by CDK1/cyclin B phosphorylation. Nucleic Acids Res..

[88] Bellé R., Derancourt J., Poulhe R., Capony J.P., Ozon R., Mulner-Lorillon O. (1989). A purified complex from Xenopus oocytes contains a p47 protein, an in vivo substrate of MPF, and a p30 protein respectively homologous to elongation factors EF-1γ and EF-1β. FEBS Lett..

[89] Kawaguchi Y., Kato K., Tanaka M., Kanamori M., Nishiyama Y., Yamanashi Y. (2003). Conserved protein kinases encoded by herpesviruses and cellular protein kinase cdc2 target the same phosphorylation site in eukaryotic elongation factor 1dekta. J. Virol..

[90] Tcherkezian J., Cargnello M., Romeo Y., Huttlin E.L., Lavoie G., Gygi S.P., Roux P.P. (2014). Proteomic analysis of cap-dependent translation identifies LARP1 as a key regulator of 5′TOP mRNA translation. Genes Dev..

[91] Aoki K., Adachi S., Homoto M., Kusano H., Koike K., Natsume T. (2013). LARP1 specifically recognizes the 3′ terminus of poly(A) mRNA. FEBS Lett..

[92] Hsu P.P., Kang S.A., Rameseder J., Zhang Y., Ottina K.A., Lim D., Peterson T.R., Choi Y., Gray N.S., Yaffe M.B. (2011). The mTOR-regulated phosphoproteome reveals a mechanism of mTORC1-mediated inhibition of growth factor signaling. Science.

[93] Yu Y., Yoon S.O., Poulogiannis G., Yang Q., Ma X.M., Villén J., Kubica N., Hoffman G.R., Cantley L.C., Gygi S.P. (2011). Phosphoproteomic analysis identifies Grb10 as an mTORC1 substrate that negatively regulates insulin signaling. Science.

[94] Hong S., Freeberg M.A., Han T., Kamath A., Yao Y., Fukuda T., Suzuki T., Kim J.K., Inoki K. (2017). LARP1 functions as a molecular switch for mTORC1-mediated translation of an essential class of mRNAs. eLife.

[95] Mura M., Hopkins T.G., Michael T., Abd-Latip N., Weir J., Aboagye E., Mauri F., Jameson C., Sturge J., Gabra H. (2015). LARP1 post-transcriptionally regulates mTOR and contributes to cancer progression. Oncogene.

[96] Xie C., Huang L., Xie S., Xie D., Zhang G., Wang P., Peng L., Gao Z. (2013). LARP1 predict the prognosis for early-stage and AFP-normal hepatocellular carcinoma. J. Transl. Med..

[97] Ye L., Lin S.T., Mi Y.S., Liu Y., Ma Y., Sun H.M., Peng Z.H., Fan J.W. (2016). Overexpression of LARP1 predicts poor prognosis of colorectal cancer and is expected to be a potential therapeutic target. Tumor Biol..

[98] Xu Z., Xu J., Lu H., Lin B., Cai S., Guo J., Zang F., Chen R. (2017). LARP1 is regulated by the XIST/miR-374a axis and functions as an oncogene in non-small cell lung carcinoma. Oncol. Rep..

